# Racial/Ethnic Disparities Among Extremely Preterm Infants in the United States From 2002 to 2016

**DOI:** 10.1001/jamanetworkopen.2020.6757

**Published:** 2020-06-10

**Authors:** Colm P. Travers, Waldemar A. Carlo, Scott A. McDonald, Abhik Das, Namasivayam Ambalavanan, Edward F. Bell, Pablo J. Sánchez, Barbara J. Stoll, Myra H. Wyckoff, Abbot R. Laptook, Krisa P. Van Meurs, Ronald N. Goldberg, Carl T. D’Angio, Seetha Shankaran, Sara B. DeMauro, Michele C. Walsh, Myriam Peralta-Carcelen, Monica V. Collins, M. Bethany Ball, Ellen C. Hale, Nancy S. Newman, Jochen Profit, Jeffrey B. Gould, Scott A. Lorch, Carla M. Bann, Margarita Bidegain, Rosemary D. Higgins

**Affiliations:** 1 Department of Pediatrics, University of Alabama at Birmingham, Birmingham; 2 Statistics and Epidemiology Unit, RTI International, Research Triangle Park, North Carolina; 3 Statistics and Epidemiology Unit, RTI International, Rockville, Maryland; 4 Department of Pediatrics, University of Iowa, Iowa City; 5 Nationwide Children’s Hospital, Department of Pediatrics, The Ohio State University, Columbus; 6 Children’s Healthcare of Atlanta, Grady Memorial Hospital, Department of Pediatrics, Emory University School of Medicine, Atlanta, Georgia; 7 Department of Pediatrics, University of Texas Southwestern Medical Center, Dallas; 8 Women and Infants’ Hospital, Department of Pediatrics, Brown University, Providence, Rhode Island; 9 Division of Neonatal and Developmental Medicine, Lucile Packard Children’s Hospital, Department of Pediatrics, Stanford University School of Medicine, Palo Alto, California; 10 Department of Pediatrics, Duke University, Durham, North Carolina; 11 University of Rochester School of Medicine and Dentistry, Rochester, New York; 12 Department of Pediatrics, Wayne State University, Detroit, Michigan; 13 The Children’s Hospital of Philadelphia, Department of Pediatrics, University of Pennsylvania, Philadelphia; 14 Rainbow Babies and Children’s Hospital, Department of Pediatrics, Case Western Reserve University, Cleveland, Ohio; 15 Eunice Kennedy Shriver National Institute of Child Health and Human Development, National Institutes of Health, Bethesda, Maryland; 16 Department of Global and Community Health, George Mason University, Fairfax, Virginia

## Abstract

**Importance:**

Racial/ethnic disparities in quality of care among extremely preterm infants are associated with adverse outcomes.

**Objective:**

To assess whether racial/ethnic disparities in major outcomes and key care practices were changing over time among extremely preterm infants.

**Design, Setting, and Participants:**

This observational cohort study used prospectively collected data from 25 US academic medical centers. Participants included 20 092 infants of 22 to 27 weeks’ gestation with a birth weight of 401 to 1500 g born at centers participating in the National Institute of Child Health and Human Development Neonatal Research Network from 2002 to 2016. Of these infants, 9316 born from 2006 to 2014 were eligible for follow-up at 18 to 26 months’ postmenstrual age (excluding 5871 infants born before 2006, 2594 infants born after 2014, and 2311 ineligible infants including 64 with birth weight >1000 g and 2247 infants with gestational age >26 6/7 weeks), of whom 745 (8.0%) did not have known follow-up outcomes at 18 to 26 months.

**Main Outcomes and Measures:**

Rates of mortality, major morbidities, and care practice use over time were evaluated using models adjusted for baseline characteristics, center, and birth year. Data analyses were conducted from 2018 to 2019.

**Results:**

In total, 20 092 infants with a mean (SD) gestational age of 25.1 (1.5) weeks met the inclusion criteria and were available for the primary outcome: 8331 (41.5%) black infants, 3701 (18.4%) Hispanic infants, and 8060 (40.1%) white infants. Hospital mortality decreased over time in all groups. The rate of improvement in hospital mortality over time did not differ among black and Hispanic infants compared with white infants (black infants went from 35% to 24%, Hispanic infants went from 32% to 27%, and white infants went from 30% to 22%; *P* = .59 for race × year interaction). The rates of late-onset sepsis among black infants (went from 37% to 24%) and Hispanic infants (went from 45% to 23%) were initially higher than for white infants (went from 36% to 25%) but decreased more rapidly and converged during the most recent years (*P* = .02 for race × year interaction). Changes in rates of other major morbidities did not differ by race/ethnicity. Death before follow-up decreased over time (from 2006 to 2014: black infants, 14%; Hispanic infants, 39%, white infants, 15%), but moderate-severe neurodevelopmental impairment increased over time in all racial/ethnic groups (increase from 2006 to 2014: black infants, 70%; Hispanic infants, 123%; white infants, 130%). Rates of antenatal corticosteroid exposure (black infants went from 72% to 90%, Hispanic infants went from 73% to 83%, and white infants went from 86% to 90%; *P* = .01 for race × year interaction) and of cesarean delivery (black infants went from 45% to 59%, Hispanic infants went from 49% to 59%, and white infants went from 62% to 63%; *P* = .03 for race × year interaction) were initially lower among black and Hispanic infants compared with white infants, but these differences decreased over time.

**Conclusions and Relevance:**

Among extremely preterm infants, improvements in adjusted rates of mortality and most major morbidities did not differ by race/ethnicity, but rates of neurodevelopmental impairment increased in all groups. There were narrowing racial/ethnic disparities in important care practices, including the use of antenatal corticosteroids and cesarean delivery.

## Key Points

**Question:**

Are racial/ethnic disparities in care practices and major outcomes increasing or decreasing among extremely preterm infants in the US?

**Findings:**

In this cohort study of 20 092 extremely preterm infants, racial/ethnic disparities in rates of antenatal corticosteroids and cesarean delivery decreased over time. Changes in rates of mortality and most major morbidities did not differ among white, black, and Hispanic infants, and while mortality decreased over time from 2002 to 2016, rates of moderate-severe neurodevelopmental impairment increased over time in all groups.

**Meaning:**

Racial/ethnic disparities in rates of potentially life-saving care practices decreased over time in the US, with reductions in mortality but increases in neurodevelopmental impairment in all racial/ethnic groups.

## Teaser

This cohort study evaluates whether racial/ethnic disparities in key care practices and major outcomes changed among extremely preterm infants from 2002 to 2016 at centers participating in the Eunice Kennedy Shriver National Institute of Child Health and Human Development Neonatal Research Network.

## Introduction

Mortality and morbidity rates among extremely preterm infants are decreasing,^1^ but few studies have focused on changes in major outcomes, including neurodevelopmental impairment, by race/ethnicity over time. Published reports document higher infant mortality rates among black infants in the United States,^2^ largely driven by a higher rate of preterm delivery.^3,4^ Although black mothers are more likely to deliver preterm and low-birth-weight infants,^5,6,7^ a survival advantage among black preterm infants and low-birth-weight infants has been reported.^8^ However, data from national population-based data sets have shown that the mortality rate among very preterm black infants is higher than that among very preterm white infants or among Hispanic infants,^4^ while the infant mortality rate may be decreasing faster among white infants and Hispanic infants relative to black infants.^2,9^

Racial disparities in health outcomes between white perterm infants and black preterm infants may be due to differences in care practices,^10,11,12^ but vital statistics databases do not collect this information.^13,14^ In addition to mortality and major morbidities, including neurodevelopmental impairment, data on selected prenatal and postnatal care practices are included in the Eunice Kennedy Shriver National Institute of Child Health and Human Development Neonatal Research Network database. The primary goal of the present study was to assess whether racial/ethnic differences in hospital mortality rates were changing over time among infants born at one of the centers in this network. We also assessed whether racial/ethnic differences in rates of major morbidities and in utilization rates of major prenatal and postnatal care practices were changing over time.

## Methods

This cohort study evaluated maternal and neonatal data collected prospectively by trained research personnel. We included infants from 22 0/7 through 27 6/7 weeks’ gestation and with a birth weight 401 g to 1500 g born at one of the centers in the National Institute of Child Health and Human Development Neonatal Research Network between January 1, 2002, and December 31, 2016. Infants with congenital anomalies who were not resuscitated and infants who were stillborn were excluded. Infants without anomalies who died during the first 12 hours after birth with or without resuscitation^15^ were included. Gestational age was determined by best obstetrical estimate when available; otherwise, the best neonatal estimate was used.^15^ We followed the Strengthening the Reporting of Observational Studies in Epidemiology (STROBE) reporting guideline. Data collection was approved by each center’s institutional review board. Most centers approved a waiver of informed consent (45 CFR part 46). Otherwise, written informed consent was obtained in a manner consistent with the US Common Rule requirements. No one received compensation or was offered any incentive for participating in this study.

### Definitions

Self-reported maternal race/ethnicity was abstracted from the medical record and used to define infant race/ethnicity. Mothers with more than 1 race, unknown race/ethnicity, or race/ethnicity other than black non-Hispanic (referred to as black in the present study), white non-Hispanic (referred to as white in the present study), or Hispanic (including black or white) were not included. Paternal race/ethnicity was not recorded. Hospital outcomes until death, hospital discharge, or 120 days were collected using standardized definitions.^1^ Bronchopulmonary dysplasia was defined as the use of supplemental oxygen at 36 weeks’ postmenstrual age. Severe intracranial hemorrhage was defined as ventricular enlargement with concurrent or prior blood in the ventricles, or parenchymal blood or echodensity or both. Proven necrotizing enterocolitis was defined as stage 2 or greater by the modified Bell criteria.^16^ Severe retinopathy of prematurity was defined as stage 3 or greater or receiving surgery or both. Moderate-severe neurodevelopmental impairment at 18 to 26 months corrected age was defined as moderate or severe cerebral palsy based on an abnormal neuromotor examination and a score higher than 2 on the Gross Motor Function Classification System scale^17^; bilateral blindness; bilateral hearing loss receiving amplification; or cognitive composite score less than 70 on Bayley Scales of Infant Development, Third Edition (Bayley-III). Infants were eligible for follow-up if they weighed 401 to 1000 g from 2006 to 2007 regardless of gestational age, if they had a gestational age from 22 0/7 to 26 6/7 weeks from 2008 to 2014 regardless of weight, or if they were in a clinical trial that assessed neurodevelopmental outcomes. For uniformity, neurodevelopmental outcomes were not analyzed for infants born before 2006 because the Bayley-III was introduced in that year.^18^ Antenatal care practices examined included exposure to antibiotics in the 72 hours before delivery, any antenatal corticosteroids, or magnesium sulfate, and mode of delivery. Postnatal care practices included treatment with surfactant, mechanical ventilation, or postnatal steroids for bronchopulmonary dysplasia, and whether any human milk was received in the first 28 days after birth.

### Statistical Analysis

The primary outcome was the difference between black infants, Hispanic infants, and white infants (the reference group) in the rate of change of the in-hospital mortality rate over time. Prespecified exploratory outcomes included changes in rates of major morbidities and receipt of care practices over time. Because mortality is a competing outcome for major morbidities in extremely preterm infants, composite rates of mortality or individual major morbidities were also evaluated.^19^ An a priori power calculation showed that the sample of all infants delivered between 2002 and 2016 would provide more than 90% power to detect a statistically significant odds ratio of 0.9 or lower or 1.1 or higher (corresponding to the primary analysis of the interaction term between race and time for in-hospital mortality), assuming a 2-sided statistical significance level of 5%.

Unadjusted differences in categorical variables were analyzed with the χ^2^ test. The Kruskal-Wallis test was used for continuous skewed variables. Logistic regression was performed adjusting for infant and maternal factors present at birth associated with adverse infant outcomes, including infant gestational age, birth weight, sex, antenatal corticosteroid exposure, mode of delivery, multiple gestation, small for gestational age (<10th centile),^20^ birth center, and prenatal care, and maternal age, educational level, insurance status, marital status, diabetes (pregestational and gestational), hypertension (chronic and pregnancy-induced), and antepartum hemorrhage. Missing data were not imputed. All models were adjusted for birth year (numerical variable) and tested for the presence of statistical interaction between birth year and race/ethnicity. A type 3 *P* value for the interaction term between race/ethnicity and time was used to evaluate whether adjusted rates of change over time for a given outcome differed by race/ethnicity. Smoothed lines were fit to the unadjusted data using a spline routine. Smoothing splines provide a flexible means for smoothing noisy data using piecewise polynomial regression modeling to uncover patterns in the data.

Post hoc logistic regression models adjusting for biological variables alone (gestational age, birth weight, sex, multiple gestation, small for gestational age, maternal diabetes, maternal hypertension, and antepartum hemorrhage), care practice variables alone (antenatal corticosteroid exposure, mode of delivery, center, and prenatal care), and socioeconomic factors alone (insurance status, maternal educational level, maternal age, and marital status) were undertaken to understand the relative association of these factors with the complete model. We conducted additional sensitivity analyses excluding maternal educational level owing to a high rate of missing data (15.9%). Sensitivity analyses were also undertaken excluding infants who died within 12 hours after birth without resuscitation to ensure that results were not affected by postnatal restriction of care and also excluding centers that were not part of the Neonatal Research Network for the entire period to examine for confounding by center participation. Differential associations of race/ethnicity by center were also explored by adding an interaction term between race/ethnicity and center.^21^ We used SAS software, version 9.4 (SAS Institute Inc), for statistical analyses, with a 2-sided *P* < .05 indicating statistical significance. Analyses were conducted from 2018 to 2019.

## Results

### Mother and Infant Characteristics

In total, 20 092 infants with a mean (SD) gestational age of 25.1 (1.5) weeks met the inclusion criteria and were available for the primary outcome: 8331 (41.5%) black infants, 3701 (18.4%) Hispanic infants, and 8060 (40.1%) white infants. Baseline maternal characteristics, including rates of documented chorioamnionitis, hypertension, antepartum hemorrhage, prenatal care, high school graduation, private health insurance, and marriage differed by race/ethnicity (Table 1). Black infants had lower mean (SD) birth weight compared with white infants (754 [197] vs 790 [208] g), whereas there was no difference in birth weights between Hispanic infants (785 [208] g) and white infants. White infants (32.2%) were more likely to be from multiple birth pregnancies compared with black infants (21.0%) or with Hispanic infants (19.6%). Of 9316 infants eligible for follow-up based on birth year, birth weight, and gestational age (excluded 5871 infants born before 2006, 2594 infants born after 2014, and 2311 ineligible infants including 64 with birth weight above 1000 g and 2247 infants with gestational age above 26 6/7 weeks), 749 (8.0%) did not have known follow-up outcomes at 18 to 26 months. Missing follow-up outcome data did not vary significantly by race/ethnicity (303 black infants [40.5%], 123 Hispanic infants [16.4%], and 323 white infants [43.1%]).

**Table 1.** Baseline Demographic and Clinical Characteristics of 20 092 Infants and Their Mothers Present at Infant Birth by Race/Ethnicity, 2002-2016

Characteristic	No. (%) of participants		
	Black	Hispanic	White
Study population, total No.	8331	3701	8060
Maternal characteristic			
Diabetes (including pregestational and gestational)	396 (4.8)	155 (4.2)	335 (4.2)
Hypertension (including chronic and pregnancy induced)	2116 (25.4)^a^	689 (18.6)^a^	1658 (20.6)
Antepartum hemorrhage	1516 (18.2)^a^	800 (21.6)	1669 (20.7)
Documented chorioamnionitis^b^	1087 (18.7)^a^	468 (18.5)	984 (16.8)
Rupture of membranes >24 h	2095 (25.9)	845 (23.6)	1938 (24.8)
Prenatal visit in this pregnancy	7688 (92.4)^a^	3480 (94.1)^a^	7807 (96.9)
Married	1947 (23.7)^a^	1732 (47.5)^a^	5101 (63.9)
Maternal or primary caretaker educational level ≥high school degree	5240 (75.4)^a^	1624 (51.9)^a^	5894 (86.4)
Maternal age, mean (SD), y	26.5 (6.3)^a^	27.4 (6.8)^a^	28.4 (6.3)
Health insurance^c^			
Medicaid	5737 (69.5)^a^	2272 (62.2)^a^	2767 (34.5)
Private insurance	2060 (25.0)^a^	766 (21.0)^a^	4935 (61.6)
Self-pay or uninsured	459 (5.6)^a^	616 (16.9)^a^	315 (3.9)
Infant characteristic			
Birth weight, mean (SD), g	754 (197)^a^	785 (208)	790 (208)
Gestational age, mean (SD), wk	25.1 (1.5)^a^	25.1 (1.5)^a^	25.2 (1.5)
Male sex	4258 (51.1)^a^	1970 (53.2)	4263 (52.9)
Multiple births	1752 (21.0)^a^	726 (19.6)^a^	2600 (32.3)
Small for gestational age	624 (7.5)	238 (6.4)	568 (7.1)

^a^ Results significant at *P* < .05, with *P* values from χ^2^ tests (categorical variables) or Kruskal-Wallis tests (continuous variables), and separately comparing black and Hispanic populations vs white population.

^b^ Data on documented chorioamnionitis were added in 2006.

^c^ Results shown for data available at birth, incorporating data at discharge or follow-up when baseline data were unavailable.

### Mortality

**Figure 1.**
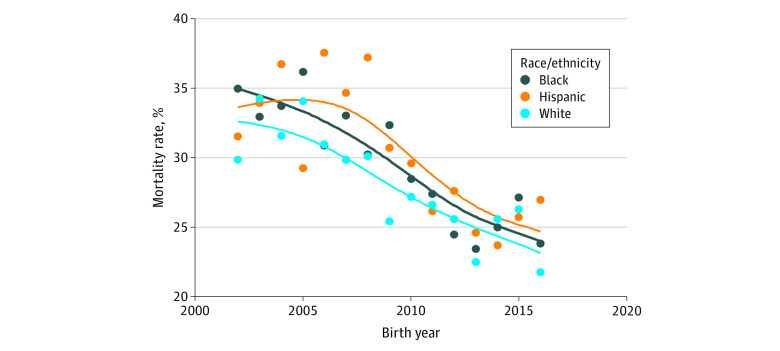
Infant Mortality Rates From 2002 to 2016 by Race/Ethnicity The rates of mortality for each race/ethnicity over time are shown using spline lines.

There was no statistically significant interaction of race by year in the adjusted rate of improved in-hospital mortality among black infants or Hispanic infants compared with white infants (*P* = .59 for race × year interaction) (Table 2; Figure 1). From 2002 to 2016, the unadjusted hospital mortality rate decreased from 35% to 24% among black infants, from 32% to 27% among Hispanic infants, and from 30% to 22% among white infants (Table 2; Figure 1). After adjustment for baseline characteristics, the hospital mortality rate decreased from 16% (95% CI, 13%-18%) to 12% (95% CI, 11%-15%) among black infants, from 17% (95% CI, 14%-21%) to 13% (95% CI, 11%-16%) among Hispanic infants, and from 21% (95% CI, 19%-24%) to 15% (95% CI, 13%-18%) among white infants (eFigure 1 in the Supplement). Black infants had significantly lower adjusted mortality rates compared with white infants from 2002 to 2011 (eFigure 1 in the Supplement). There was no significant difference in adjusted mortality rates between Hispanic and white infants for any study year.

**Table 2.** Percentage Change in Rate of Death or Major Composite Outcomes From First Year of Data Studied (2002) to Last Year of Data Studied (2016)

Outcome	Black infants					Hispanic infants						White infants			*P* value for race × year interaction		
	Rate, %		% Change	*P* value vs white group		Rate, %		% Change	*P* value vs white group			Rate, %		% Change	Overall type 3	Black vs white	Hispanic vs white
	First year	Last year		First year	Last year	First year	Last year		First year		Last year	First year	Last year				
Death before discharge	35	24	−32	<.001^a^	.02^a^	32	27	−14	.03^a^	.22		30	22	−27	.59	.31	.62
Death excluding infants not resuscitated^b^	27	21	−25	<.001^a^	.09	25	20	−21	.04^a^	.17		27	18	−32	.44	.21	.74
Traditional BPD or death	61	59	−4	<.001^a^	<.001^a^	61	63	3	.10	<.001^a^		66	77	16	.14	.20	.06
Severe ICH, PVL, or death	46	35	−24	.01^a^	.01^a^	42	36	−15	.39	.39		40	34	−13	.99	.97	.97
Severe ROP or death	49	33	−32	<.001^a^	<.001^a^	50	39	−22	.94	.03^a^		46	36	−22	.33	.24	.18
Proven NEC or death	42	32	−25	<.001^a^	.42	37	31	−16	.004^a^	.56		35	27	−22	.21	.11	.18
Late-onset sepsis or death	59	40	−32	.04^a^	.002^a^	62	42	−32	.20	.006^a^		54	40	−25	.04^a^	.51	.01^a^
NDI or death at follow-up, 2006-2014^c^	46	47	2	<.001^a^	.05	52	45	−14	.44	.15		44	46	5	.09	.33	.16
Death at follow up, 2006-2014^c^	38	33	−14	.004	.12	47	28	−39	.76	.004		39	33	−15	.02^a^	.37	.04^a^

Abbreviations: BPD, bronchopulmonary dysplasia; ICH, intracranial hemorrhage; LOS, late-onset sepsis; NDI, neurodevelopmental impairment; NEC, necrotizing enterocolitis; PVL, periventricular leukomalacia; ROP, retinopathy of prematurity; SGA, small for gestational age.

^a^ Significant at *P* < .05. The *P* values for mortality and composite outcomes, including death or traditional BPD, death or severe ICH or PVL, death or severe ROP, death or proven NEC, death or LOS, and death or moderate to severe NDI, are estimated with the white group used as the referent category. Models include race/ethnicity (black non-Hispanic, Hispanic, white non-Hispanic), year of birth, gestational age, birth weight, maternal age, male sex, antenatal steroids, cesarean delivery, multiple birth, SGA, center, marital status, maternal educational level, insurance status, diabetes (including pregestational and gestational), hypertension (including chronic and pregnancy induced), antepartum hemorrhage, and prenatal care.

^b^ After exclusion of 1321 infants (529 black, 452 white, and 340 Hispanic infants) who died without resuscitation within 12 hours after birth.

^c^ Does not include data for 5871 infants born before 2006, 2594 infants born after 2014, or 2311 ineligible infants, which includes 64 with birth weight greater than 1000 g and 2247 infants with gestational age greater than 26 6/7 weeks.

There were 1321 infants who died within 12 hours without resuscitation. The racial/ethnic distribution was 529 of 8331 black infants (6.3%), 340 of 3701 Hispanic infants (9.2%), and 452 of 8060 white infants (5.6%) (*P* < .001). After exclusion of these infants, there remained no difference in the rate of improvement in hospital mortality among black infants (went from 27% to 21%) and Hispanic infants (went from 25% to 20%) compared with white infants (went from 27% to 18%) over time (*P* = .44, for race × year interaction) (Table 2; eFigure 2 in the Supplement). Among those eligible for follow-up at 22 to 26 months, mortality decreased over time across all groups, and there was a significant race × year interaction (*P* = .02) due to a faster decrease among Hispanic infants (went from 47% to 28%) compared with white infants (went from 39% to 33%) during this shortened period (Table 2).

### Mortality or Individual Major Morbidities

For composite outcomes, there was a significant race × year interaction in the rate of late-onset sepsis or death because the rates among black infants (went from 59% to 40%) and Hispanic infants (went from 62% to 42%) were initially higher but decreased faster compared with white infants (went from 54% to 40%) and converged during the most recent years (*P* = .04; Table 2). Race × year interactions were not found for other composite outcomes, including moderate-severe neurodevelopmental impairment or death; bronchopulmonary dysplasia or death; severe intracranial hemorrhage or periventricular leukomalacia or death; proven necrotizing enterocolitis or death; and severe retinopathy of prematurity or death.

### Major Morbidities

**Figure 2.**
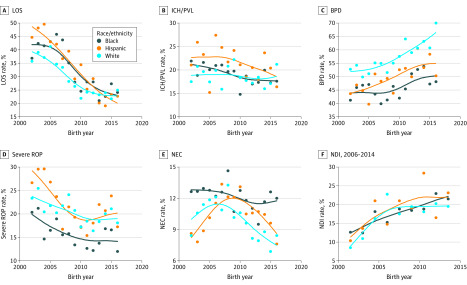
Selected Major Morbidity Rates Among Infants by Race/Ethnicity Over Time The rate of morbidities for each race/ethnicity over time are shown using spline lines. BPD indicates bronchopulmonary dysplasia; ICH/PVL, intracranial hemorrhage/periventricular leukomalacia; LOS, late-onset sepsis; NEC, necrotizing enterocolitis; NDI, neurodevelopmental impairment; ROP, retinopathy of prematurity.

For individual morbidity outcomes, there was a significant race × year interaction in the rate of late-onset sepsis because the rates among black infants (went from 37% to 24%) and Hispanic infants (went from 45% to 23%) were initially higher but decreased faster compared with white infants (went from 36% to 25%) and converged during the most recent years (*P* = .02) (Table 3; Figure 2). Race × year interactions were not found for other morbidity outcomes, including moderate-severe neurodevelopmental impairment, bronchopulmonary dysplasia, severe intracranial hemorrhage or periventricular leukomalacia, proven necrotizing enterocolitis, and severe retinopathy of prematurity. Rates of moderate-severe neurodevelopmental impairment increased over time across all groups (increase from 2006 to 2014: black infants, 70%; Hispanic infants, 123%; white infants, 130%).

**Table 3.** Percentage Change in Rate of Major Morbidities From First Year to Last Year Available

Outcome	Black infants					Hispanic infants						White infants			*P* value for race × year interaction		
	Rate, %		% Change	*P* value vs white		Rate, %		% Change	*P* value vs white			Rate, %		% Change	Overall type 3	Black vs white	Hispanic vs white
	First year	Last year		First year	Last year	First year	Last year		First year		Last year	First year	Last year				
Traditional BPD	41	48	17	<.001^a^	<.001^a^	44	50	15	.16	<.001^a^		53	70	33	.24	.23	.11
Severe ICH or PVL	22	18	−20	.62	.05	21	16	−22	.54	.66		18	21	21	.62	.36	.93
Severe ROP	20	12	−41	<.001^a^	<.001^a^	27	17	−35	.43	.23		23	18	−22	.10	.04^a^	.21
Proven NEC	13	12	−5	.98	.06	9	8	−12	.17	.31		8	8	0	.29	.24	.14
Late-onset sepsis	37	24	−35	.79	.04^a^	45	23	−49	.004^a^	.08		36	25	−30	.02^a^	.24	.005^a^
NDI, 2006-2014^b^	13	22	70	.08	.13	10	23	123	.25	.52		9	20	130	.83	.76	.72

Abbreviations: BPD, bronchopulmonary dysplasia; ICH, intracranial hemorrhage; LOS, late-onset sepsis; NDI, neurodevelopmental impairment; NEC, necrotizing enterocolitis; PVL, periventricular leukomalacia; ROP, retinopathy of prematurity; SGA, small for gestational age.

^a^ Significant at *P* < .05. The *P* values for morbidities, including traditional BPD, severe ICH or PVL, severe ROP, proven NEC, LOS, and moderate to severe NDI, are estimated with the white group used as the referent category. Models include race/ethnicity (black non-Hispanic, Hispanic, and white non-Hispanic), year of birth, gestational age, birth weight, maternal age, male sex, antenatal steroids, cesarean delivery, multiple birth, SGA, center, marital status, maternal educational level, insurance status, diabetes (including pregestational and gestational), hypertension (including chronic and pregnancy induced), antepartum hemorrhage, and prenatal care.

^b^ Does not include data for 5871 infants born before 2006, 2594 infants born after 2014, or 2311 ineligible infants, which includes 64 with birth weight greater than 1000 g and 2247 infants with gestational age greater than 26 6/7 weeks.

### Care Practices

There was a significant race × year interaction for exposure to any antenatal corticosteroids because the rate was initially lower among black infants (went from 72% to 90%) and Hispanic infants (went from 73% to 83%), but this difference with white infants (went from 86% to 90%) decreased over time (*P* = .01 for race × year interaction) (eTable 1 and eFigure 3 in the Supplement). Differences in cesarean delivery narrowed significantly over time as the rate increased among black and Hispanic infants compared with white infants (*P* = .03 for race × year interaction). Increasing rates of antibiotic exposure within 72 hours before delivery were observed in all groups but increased faster among Hispanic infants (*P* = .01 for race × year interaction). There was no difference in the rate of treatment with magnesium sulfate before delivery by race/ethnicity. The rate of treatment with mechanical ventilation decreased across all groups over time, and there was a significant race × year interaction because rates of treatment decreased faster among Hispanic infants (black infants went from 91% to 88%, Hispanic infants went from 95% to 83%, and white infants 94% to 90%; *P* = .02 for race × year interaction) (eTable 1 and eFigure 3 in the Supplement). There was no significant race × year interaction for treatment with surfactant or postnatal corticosteroids for bronchopulmonary dysplasia or for breast milk exposure in the first 28 days after birth.

### Sensitivity Analyses

Center participation was examined as a potential confounder in an analysis restricted to 11 centers that participated for the entire study period, and the results for mortality over time did not differ (*P* = .79 for race × year interaction). To better understand center differences by race/ethnicity, we then dropped the race × year interaction term and added a term for race × center interaction to allow for race/ethnicity associations to differ by center. This interaction was statistically significant (*P* < .001). However, further analyses showed that this race × center interaction was mostly due to differences in magnitude rather than to the direction of the association because the trend toward lower adjusted mortality rates among minority infants was generally consistent (eFigure 4 in the Supplement). There was a high degree of collinearity between race/ethnicity and differences in socioeconomic factors, perinatal care practices, and biological factors, which made it difficult to determine the relative contribution of each variable to any differences in outcomes (eTable 2 in the Supplement). Sensitivity analyses excluding maternal educational level did not significantly alter our results.

## Discussion

This large multicenter cohort study showed no racial/ethnic disparities in changes over time in mortality or most major neonatal morbidities among extremely preterm infants. While hospital mortality decreased over time, rates of moderate-severe neurodevelopmental impairment increased across all racial/ethnic groups. Racial/ethnic disparities in late-onset sepsis narrowed over time, decreasing faster among black infants and Hispanic infants compared with white infants before converging. Racial/ethnic disparities in the rates of important care practices, including receipt of antenatal corticosteroids and cesarean delivery, also decreased over time among black infants and Hispanic infants compared with white infants.

Population-based data sets comparing survival trends have noted a narrowing survival advantage among extremely preterm and extremely low-birth-weight black infants compared with white infants,^22,23,24^ and 1 study found that mortality increased among very low-birth-weight black infants and was associated with a survival disadvantage.^25^ Recent data from the Vermont Oxford Network noted a narrowing survival disadvantage among black and among Hispanic very preterm infants.^12^ The aforementioned studies did not adjust for many known risk factors for adverse outcomes, including biological, care practice, and socioeconomic variables.^26^ In the present study, in contrast to the unadjusted mortality rates, adjusted mortality rates favored black infants and Hispanic infants compared with white infants.

Exclusion of infants who died without resuscitation did not alter our results, suggesting that mortality rates by race/ethnicity were not significantly associated with postnatal restriction of care. However, a larger proportion of Hispanic infants died without resuscitation. Differences in hospital rates of active treatment account for a large proportion of variation in survival among periviable infants, and these rates vary by race/ethnicity.^21^ Socioeconomic factors are associated with lower rates of survival within 1 hour after birth among periviable infants, likely owing to differences in providing resuscitation.^27^ We found multiple socioeconomic differences by race/ethnicity, but it is unknown whether these influenced clinical decision-making.

A significant proportion of racial/ethnic disparities in infant outcomes may be attributed to birth center.^28^ Racial/ethnic disparities in the quality of care and infant outcomes among preterm infants vary between centers.^11,28^ Our data represented a nonrandom sample of extremely preterm infants from a geographically diverse set of academic centers of excellence. Although the center was a significant confounder for overall mortality by race/ethnicity in the present study, the direction of this association indicated a trend toward lower adjusted mortality rates among minority infants across most centers.

Using a database that overcomes the limitations of vital statistics data sets,^13,14^ the Vermont Oxford Network found narrowing disparities in late-onset sepsis, receipt of antenatal corticosteroids, and cesarean delivery.^12^ Disproportionate improvements in rates of late-onset sepsis and antenatal corticosteroid receipt^29^ among black infants and Hispanic infants were also identified in our study, suggesting that minority infants benefitted most from quality improvement initiatives. Willingness to perform cesarean delivery is associated with improved survival,^30^ and narrowing disparities in cesarean delivery may reflect improved fetal monitoring. Moderate-severe neurodevelopmental impairment increased over time across all groups in our study, emphasizing the importance of long-term follow-up data. It is possible that this increase was associated with improvements in mortality because there was no significant change in the rate of death or moderate-severe neurodevelopmental impairment.

### Limitations

This study analyzed major outcomes, including neurodevelopmental impairment, using a large prospective database with adjustment for multiple biological, care practice, and socioeconomic variables. However, there were a number of limitations that must be noted. Race/ethnicity was recorded as reported by the mother alone, although self-reported maternal race/ethnicity and infant genetic ancestry are closely correlated.^31^ Furthermore, genetic ancestry markers show much heterogeneity,^32^ indicating that race/ethnicity may be largely a social construct.^33^ It is unlikely that our results are due solely to confounding, but there was likely residual bias in our models.^33^ For example, data were unavailable on the adequacy of prenatal care, fetal monitoring, perinatal decision-making and counseling, the length of prenatal maternal admission,^30,34,35^ and parental employment. The multiple testing with a 5% significance level may have resulted in outcomes being significant by chance. Our detailed data set includes a subset of infants born in academic centers in the United States that may not be representative of the broader population.

## Conclusions

This cohort study found no racial/ethnic differences in changes in mortality rates or most major morbidities among extremely preterm infants, with mortality decreasing over time across all groups. Racial/ethnic disparities in the rate of late-onset sepsis decreased over time. There were narrowing racial/ethnic disparities in key interventions, including receipt of antenatal corticosteroids and cesarean delivery. However, there were increasing rates of moderate-severe neurodevelopmental impairment that did not differ by race/ethnicity. Adherence to evidence-based medicine and continued quality improvement efforts should further improve outcomes of extremely preterm infants among all racial and ethnic groups.

## Notes

Supplement. **eTable 1.** Selected Antenatal and Postnatal Treatments by Race/Ethnicity **eTable 2.** Comparison of Effect Sizes for Differences in Type 3 *P* Values Between Different Models **eFigure 1.** Adjusted Mortality Rate Over Time by Race/Ethnicity (2002-2016) Comparing Black With White Infants and Hispanic With White Infants **eFigure 2.** Mortality by Race/Ethnicity Over Time (2002-2016) Excluding 1321 Infants (529 Black, 452 White, and 340 Hispanic) Who Died Within 12 Hours Without Delivery Room Resuscitation **eFigure 3.** Selected Postnatal and Antenatal Care Practices by Race/Ethnicity Over Time **eFigure 4.** Adjusted Odds Ratios and 95% Confidence Intervals Comparing Mortality for Black vs White Infants and Hispanic vs White Infants at Each of the 25 Centers That Participated
