# Supplementary material for: Racial/Ethnic Disparities Among Extremely Preterm Infants in the United States From 2002 to 2016
Source: JAMA Netw Open. 2020 Jun 10;3(6):e206757. doi: 10.1001/jamanetworkopen.2020.6757 (PMC7287569; doi:10.1001/jamanetworkopen.2020.6757)
Supplement: Supplement. — eTable 1. Selected Antenatal and Postnatal Treatments by Race/Ethnicity eTable 2. Comparison of Effect Sizes for Differences in Type 3 P Values Between Different Models eFigure 1. Adjusted Mortality Rate Over Time by Race/Ethnicity (2002-2016) Comparing Black With White Infants and Hispanic With White Infants eFigure 2. Mortality by Race/Ethnicity Over Time (2002-2016) Excluding 1321 Infants (529 Black, 452 White, and 340 Hispanic) Who Died Within 12 Hours Without Delivery Room Resuscitation eFigure 3. Selected Postnatal and Antenatal Care Practices by Race/Ethnicity Over Time eFigure 4. Adjusted Odds Ratios and 95% Confidence Intervals Comparing Mortality for Black vs White Infants and Hispanic vs White Infants at Each of the 25 Centers That Participated [file jamanetwopen-3-e206757-s001.pdf]

Supplementary Online Content

Travers CP, Carlo WA, McDonald SA, et al; Generic Database and Follow-up Subcommittees of the Eunice Kennedy Shriver National Institute of Child Health and Human Development Neonatal Research Network. Racial/ethnic disparities among extremely preterm infants in the United States from 2002 to 2016. *JAMA Netw Open*. 2020;3(6):e206757. doi:10.1001/jamanetworkopen.2020.6757

eTable 1. Selected Antenatal and Postnatal Treatments by Race/Ethnicity

eTable 2. Comparison of Effect Sizes for Differences in Type 3 *P* Values Between Different Models

eFigure 1. Adjusted Mortality Rate Over Time by Race/Ethnicity (2002-2016) Comparing Black With White Infants and Hispanic With White Infants

efigure 2. Mortality by Race/Ethnicity Over Time (2002-2016) Excluding 1321 Infants (529 Black, 452 White, and 340 Hispanic) Who Died Within 12 Hours Without Delivery Room Resuscitation

eFigure 3. Selected Postnatal and Antenatal Care Practices by Race/Ethnicity Over Time

eFigure 4. Adjusted Odds Ratios and 95% Confidence Intervals Comparing Mortality for Black vs White Infants and Hispanic vs White Infants at Each of the 25 Centers That Participated

This supplementary material has been provided by the authors to give readers additional information about their work.

© 2020 Travers CP et al. *JAMA Network Open*.

eTable 1. Selected Antenatal and Postnatal Treatments by Race/Ethnicity

Outcome	Black					Hispanic					White			Overall Type 3 p-value for Race-Year interaction	P-value for Race-Year interaction black v white	P-value for Race-Year interaction Hispanic v white
	First year	Last year	% change	P-value for black v white First year	P-value for black v white Last year	First year	Last year	% change	P-value for Hispanic v white First year	P-value for Hispanic v white Last year	First year	Last year	% change			
Antenatal corticosteroid exposure	72%	90%	24%	<0.001*	0.44	73%	83%	13%	0.19	0.32	86%	90%	4%	0.01*	0.009*	0.92
Antenatal magnesium sulfate exposure, 2011-2016	69%	80%	17%	0.04*	0.17	62%	77%	24%	0.56	0.99	73%	83%	13%	0.92	0.77	0.70
Antenatal antibiotics within 72 hours of delivery, 2006-2016	59%	71%	19%	<0.001*	0.31	58%	80%	38%	0.16	0.71	59%	74%	25%	0.01*	0.003*	0.56
Delivery by cesarean section	45%	59%	30%	0.005*	0.24	49%	59%	20%	0.01*	0.06	62%	63%	1%	0.03*	0.33	0.007*
Surfactant use	83%	82%	-1%	<0.001*	0.08	82%	79%	-4%	0.57	0.10	89%	86%	-4%	0.25	0.31	0.50
Mechanical ventilation	91%	88%	-4%	0.03*	<0.001*	95%	83%	-13%	0.29	<0.001*	94%	90%	-4%	0.02*	0.63	0.006*
Treatment with postnatal steroids for BPD	18%	25%	39%	0.18	0.28	16%	20%	25%	0.02*	0.41	23%	26%	15%	0.63	0.84	0.34
Received breast milk within first 28 days, 2006-2016	72%	96%	34%	0.02*	0.72	89%	97%	9%	<0.001*	0.27	90%	98%	8%	0.06	0.15	0.28

Results are significant with a p-value < 0.05. P-values are estimated with the white group used as the referent category. Models include: race/ethnicity [black non-Hispanic, Hispanic, white non-Hispanic], year of birth, gestational age, birth weight, mother's age, male sex, antenatal steroids, C-section, multiple birth, SGA, center, marital status, maternal education, insurance status, diabetes (including pre-gestational and gestational), hypertension (including chronic and pregnancy induced), antepartum hemorrhage, and prenatal care.

© 2020 Travers CP et al. *JAMA Network Open*.

eTable 2. Comparison of Effect Sizes for Differences in Type 3 *P* Values Between Different Models

Outcome	Type 3 p-value for race-year interaction (unadjusted)<br><br>Beta estimates for black and Hispanic	Type 3 p-value for race-year interaction (adjusted for biological factors[a])<br><br>Beta estimates for black and Hispanic	Type 3 p-value for race-year interaction (adjusted for care practices[b])<br><br>Beta estimates for black and Hispanic	Type 3 p-value for race-year interaction (adjusted for socioeconomic factors[c])<br><br>Beta estimates for black and Hispanic	Type 3 p-value for race-year interaction (fully adjusted)<br><br>Beta estimates for black and Hispanic
Death before discharge	P= 0.77<br>β= -0.005 and 0.001	P= 0.44<br>β= -0.006 and 0.009	P= 0.33<br>β= 0.002 and -0.01	P= 0.44<br>β= 0.005 and 0.02	P= 0.59<br>β= 0.01 and 0.007
Death excluding infants not resuscitated	P= 0.82<br>β= -0.002 and -0.007	P= 0.81<br>β= -0.004 and 0.004	P= 0.12<br>β= 0.002 and -0.02	P= 0.34<br>β= 0.01 and 0.01	P= 0.44<br>β= 0.01 and 0.005
Traditional BPD or death	P= 0.001*<br>β= -0.03 and -0.01	P= 0.0002*<br>β= -0.03 and -0.009	P= 0.04*<br>β= -0.009 and -0.03	P= 0.01*<br>β= -0.02 and -0.003	P=0.14<br>β= -0.01 and -0.02
Severe ICH, PVL, or death	P= 0.41<br>β= -0.10 and -0.003	P= 0.36<br>β= -0.01 and 0.002	P= 0.39<br>β= -0.001 and -0.01	P= 0.46<br>β= -0.006 and 0.007	P= 0.998<br>β= 0.0004 and -0.0004
Severe ROP or death	P= 0.57<br>β= -0.007 and -0.008	P= 0.56<br>β= -0.01 and -0.005	P= 0.09<br>β= -0.009 and -0.02	P= 0.997<br>β= -0.0007 and -0.0001	P= 0.33<br>β= -0.01 and -0.02
Proven NEC or death	P= 0.52<br>β= 0.0008 and 0.01	P= 0.20<br>β= 0.002 and 0.02	P= 0.53<br>β= 0.007 and -0.004	P= 0.054<br>β= 0.01 and 0.03	P= 0.21<br>β= 0.02 and 0.02
Late-onset sepsis or death	P= 0.03*<br>β= -0.01 and -0.02	P= 0.04*<br>β= -0.01 and -0.03	P= 0.002*<br>β= -0.007 and -0.03	P= 0.16<br>β= -0.007 and -0.02	P= 0.04*<br>β= -0.006 and -0.03
NDI or death, 2006-2014	P= 0.19<br>β= -0.01 and -0.04	P= 0.23<br>β= -0.01 and -0.05	P= 0.04*<br>β= -0.003 and -0.06	P= 0.16<br>β= 0.02 and -0.02	P= 0.09<br>β= 0.02 and -0.04
Traditional BPD	P= 0.02*<br>β= -0.02 and -0.008	P= 0.002*<br>β= -0.03 and -0.001	P= 0.16<br>β= -0.009 and -0.02	P= 0.054<br>β= -0.02 and -0.004	P= 0.24<br>β= -0.01 and -0.02
Severe ICH or PVL	P= 0.32<br>β= -0.01 and -0.01	P= 0.53<br>β= -0.01 and -0.002	P= 0.49<br>β= -0.006 and -0.02	P= 0.31<br>β= -0.02 and -0.002	P= 0.62<br>β= -0.01 and -0.001
Severe ROP	P= 0.44<br>β= -0.006 and -0.02	P= 0.68<br>β= -0.01 and -0.001	P= 0.07<br>β= -0.02 and -0.03	P= 0.61<br>β= -0.004 and -0.01	P= 0.10<br>β= -0.03 and -0.02
Proven NEC	P= 0.12<br>β= 0.02 and 0.03	P= 0.10<br>β= 0.02 and 0.03	P= 0.36<br>β= 0.02 and 0.02	P= 0.049*<br>β= 0.02 and 0.04	P= 0.29<br>β= 0.02 and 0.03
Late-onset sepsis	P= 0.01*<br>β= -0.01 and -0.03	P= 0.07<br>β= -0.01 and -0.03	P= 0.005*<br>β= -0.01 and -0.04	P= 0.01*<br>β= -0.01 and -0.03	P= 0.02*<br>β= -0.01 and -0.04
NDI, 2006-2014	P= 0.98	P= 0.93	P= 0.90	P= 0.99	P= 0.83

© 2020 Travers CP et al. *JAMA Network Open*.

	β= -0.004 and 0.004	β= -0.01 and -0.0004	β= 0.01 and -0.005	β= 0.002 and 0.004	β= 0.01 and -0.02
Antenatal corticosteroid exposure	P= 0.001*<br>β= 0.02 and -0.02	P= 0.0008*<br>β= 0.03 and -0.02	P= 0.02*<br>β= 0.02 and -0.003	P= 0.0009*<br>β= 0.02 and -0.02	P= 0.01*<br>β= 0.03 and 0.002
Antenatal magnesium sulfate, 2011-2016	P= 0.98<br>β= 0.008 and 0.005	P= 0.80<br>β= 0.02 and 0.03	P= 0.96<br>β= -0.004 and -0.02	P= 0.94<br>β= 0.01 and 0.01	P= 0.92<br>β= 0.01 and 0.02
Antenatal antibiotics within 72 hours of delivery, 2006-2016	P= 0.25<br>β= -0.02 and 0.01	P= 0.26<br>β= -0.01 and 0.02	P= 0.01*<br>β= -0.04 and -0.004	P= 0.07<br>β= -0.03 and -0.0001	P= 0.01*<br>β= -0.05 and -0.01
Delivery by cesarean section	P= 0.09<br>β= 0.01 and 0.02	P= 0.20<br>β= 0.001 and 0.02	P= 0.045*<br>β= 0.01 and 0.02	P= 0.09<br>β= 0.01 and 0.02	P= 0.03*<br>β= 0.009 and 0.03
Surfactant use	P= 0.20<br>β= 0.02 and 0.008	P= 0.23<br>β= 0.02 and 0.02	P= 0.052<br>β= 0.01 and -0.02	P= 0.32<br>β= 0.02 and 0.01	P= 0.25<br>β= 0.01 and -0.01
Mechanical ventilation	P= 0.06<br>β= -0.004 and -0.04	P= 0.06<br>β= -0.007 and -0.05	P= 0.002*<br>β= -0.008 and -0.07	P= 0.20<br>β= -0.002 and -0.03	P= 0.02*<br>β= -0.008 and -0.06
Treatment with postnatal steroids for BPD	P= 0.04*<br>β= 0.03 and 0.01	P= 0.03*<br>β= 0.03 and 0.03	P= 0.57<br>β= 0.01 and 0.01	P= 0.19<br>β= 0.02 and 0.009	P= 0.63<br>β= 0.002 and 0.02
Received breast milk within first 28 days, 2006-2016	P= 0.01*<br>β= 0.07 and -0.01	P= 0.01*<br>β= 0.07 and -0.01	P= 0.09<br>β= 0.04 and -0.04	P= 0.02*<br>β= 0.07 and -0.006	P= 0.06<br>β= 0.04 and -0.05

*Results are significant with a p-value < 0.05

[a]Biological model variables: Gestational age, birth weight, sex, multiple gestation, small for gestational age (less than the 10th centile), maternal diabetes (including pre-gestational and gestational), hypertension (including chronic and pregnancy induced), and antepartum hemorrhage

[b]Care practice model variables: Antenatal corticosteroid exposure, mode of delivery, center, and prenatal care

[c]Socioeconomic status model variables: Insurance status, maternal education, maternal age, marital status

© 2020 Travers CP et al. *JAMA Network Open*.

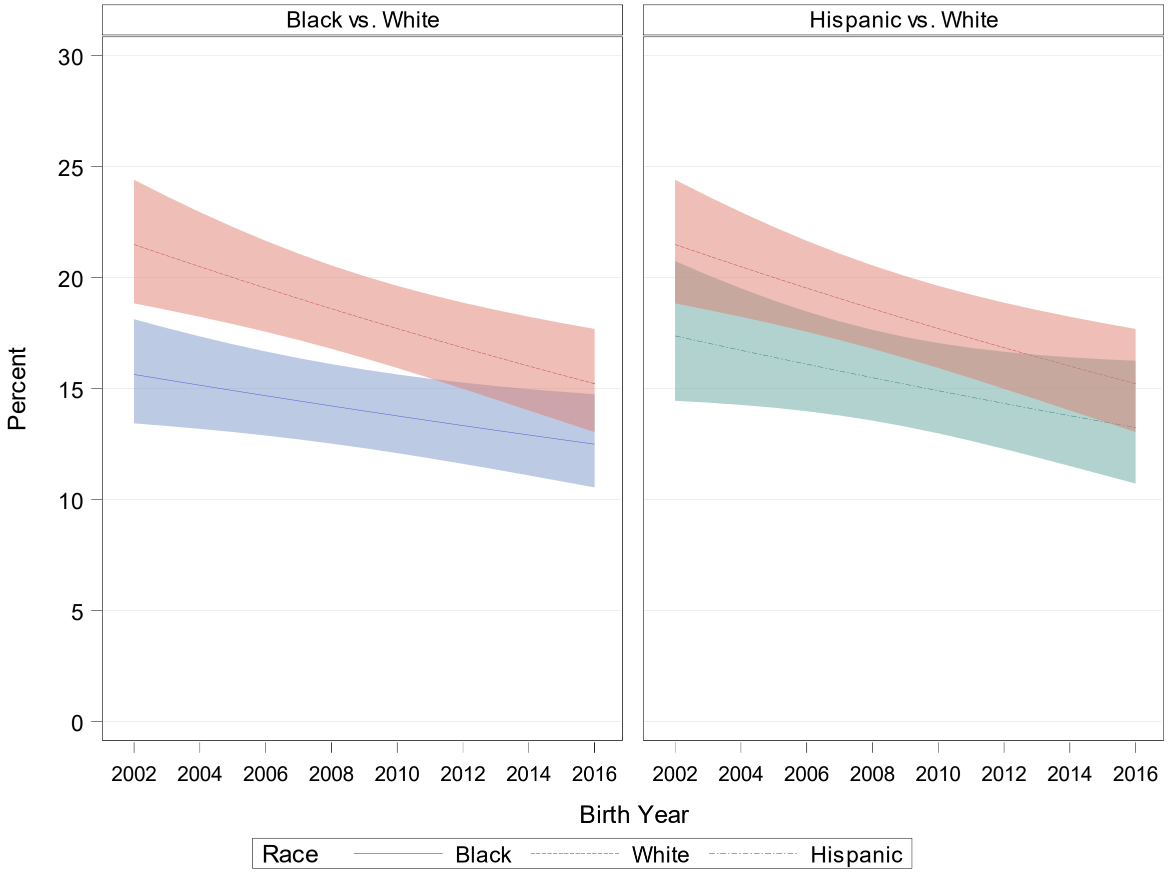

eFigure 1. Adjusted Mortality Rate Over Time by Race/Ethnicity (2002-2016) Comparing Black With White Infants and Hispanic With White Infants. Adjusted mortality rates over time are shown with 95% confidence intervals. Predicted probabilities were generated using fully adjusted regression models. Black infants had lower adjusted mortality rates compared with white infants from 2002 to 2011. There was no difference in adjusted mortality rates between Hispanic and white infants from 2002 to 2016. Improvements in mortality rates over time did not differ by race/ethnicity (p=0.59 for the year by race interaction).

© 2020 Travers CP et al. *JAMA Network Open*.

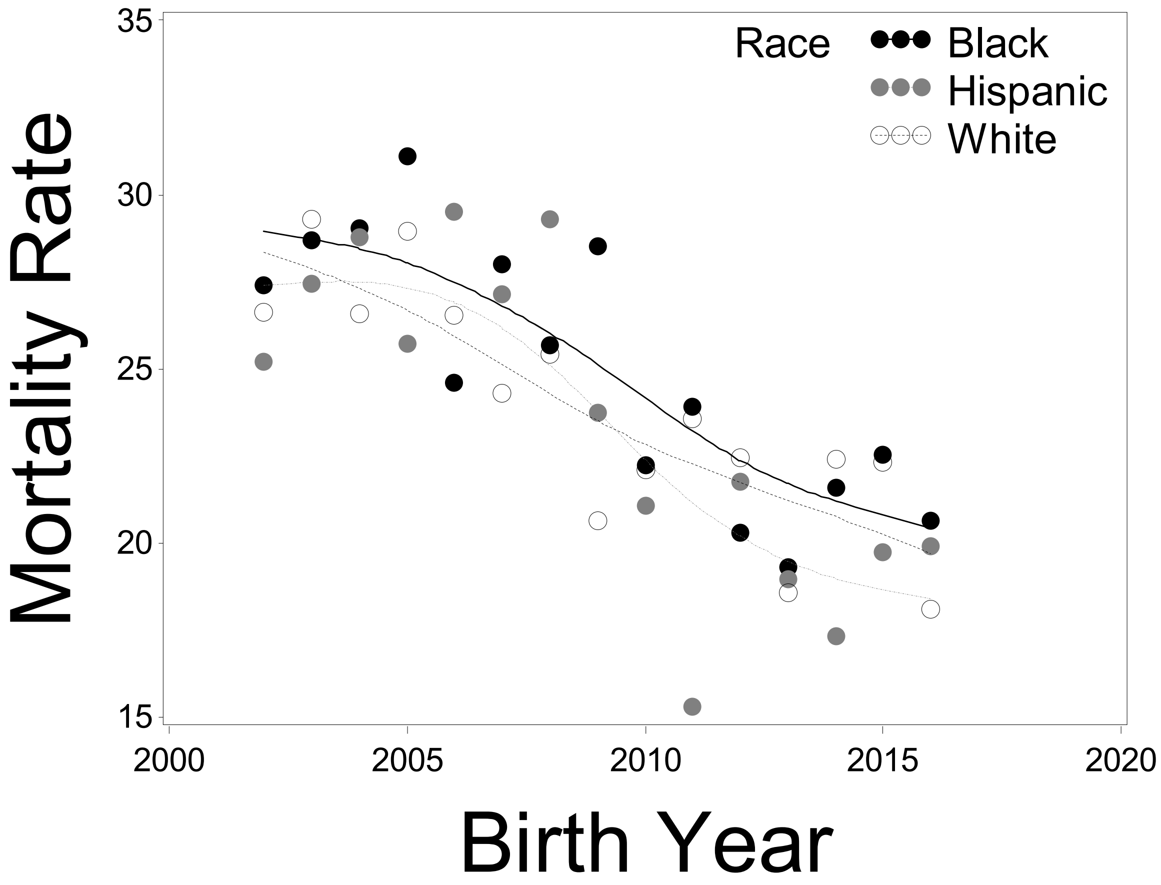

efigure 2. Mortality by Race/Ethnicity Over Time (2002-2016) Excluding 1321 Infants (529 Black, 452 White, and 340 Hispanic) Who Died Within 12 Hours Without Delivery Room Resuscitation. The proportion of infants in relation to the complete cohort was 6.3% of black infants, 9.2% of Hispanic infants, and 5.6% of white infants ($p<0.001$). Mortality rates for each race and ethnicity over time are shown using spline lines. Improvements in mortality rates over time did not differ by race/ethnicity ($p=0.44$ for the year by race interaction).

© 2020 Travers CP et al. *JAMA Network Open*.

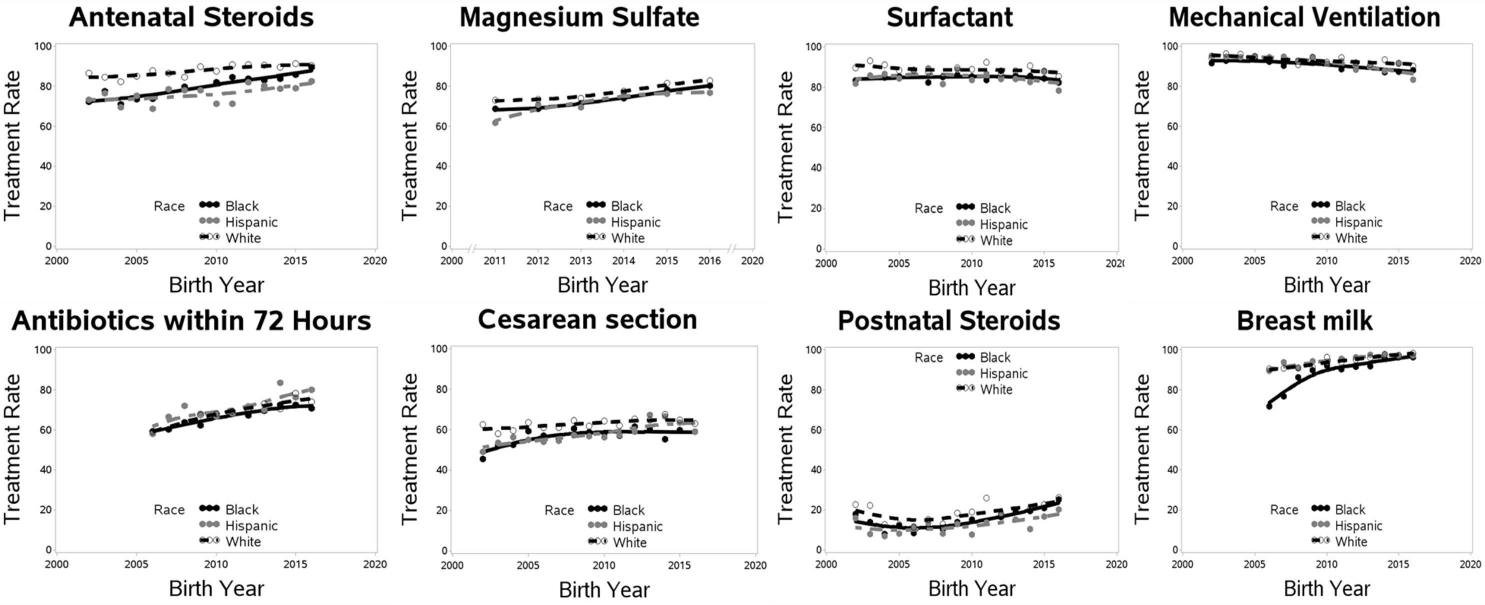

eFigure 3. Selected Postnatal and Antenatal Care Practices by Race/Ethnicity Over Time. The rate of antenatal care practices for each race and ethnicity over time are shown using spline lines. There was a significant race-year interaction for rates of exposure to antenatal corticosteroids (p=0.01), antenatal antibiotics within 72 hours of delivery (p=0.01), and delivery by cesarean section (p=0.03). There was a significant race-year interaction for treatment with mechanical ventilation (p=0.02). There was no significant race-year interaction for antenatal exposure to magnesium sulfate, treatment with surfactant, treatment with postnatal steroids for bronchopulmonary dysplasia, or the receipt of breast milk in the first 28 days after birth.

© 2020 Travers CP et al. *JAMA Network Open*.

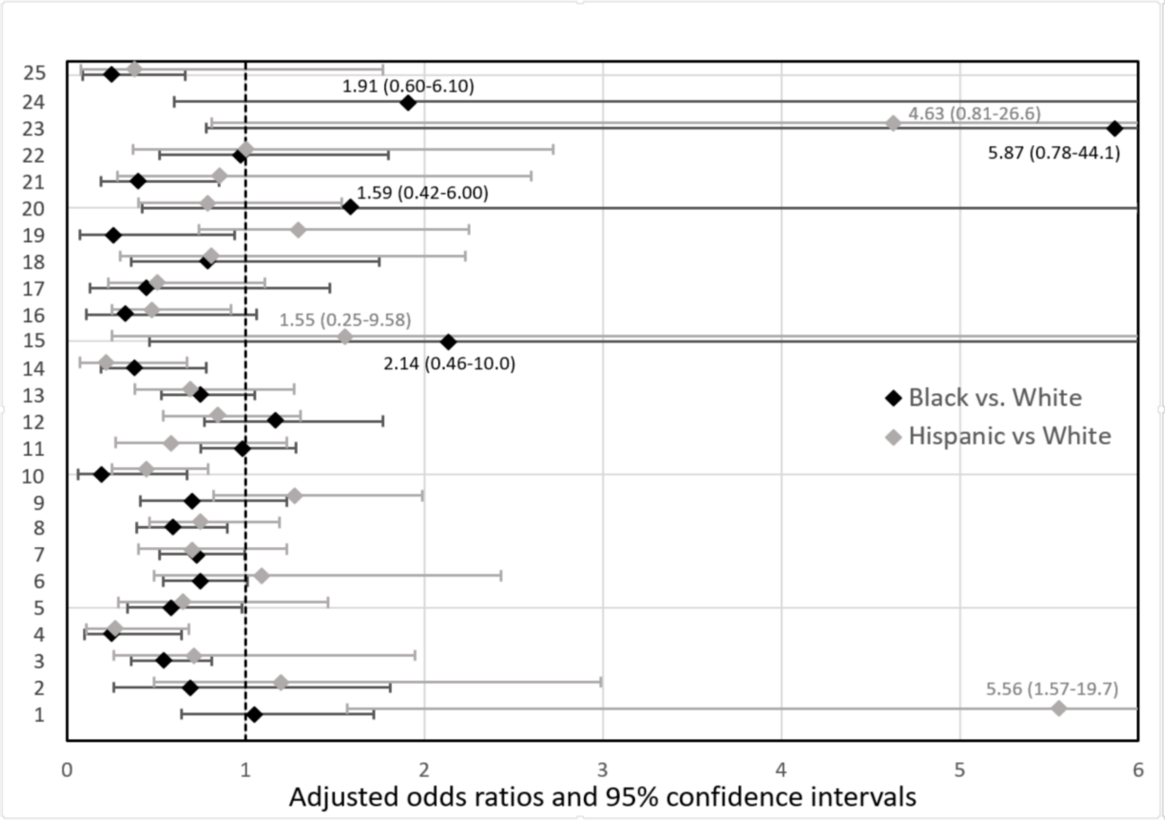

eFigure 4. Adjusted Odds Ratios and 95% Confidence Intervals Comparing Mortality for Black vs White Infants and Hispanic vs White Infants at Each of the 25 Centers That Participated. The direction of the race-center effect was largely positive indicating a trend towards lower adjusted mortality rates among black and Hispanic infants compared with white infants across the majority of centers. There were no Hispanic infants at center 24 meeting the study entry criteria over the study period.

© 2020 Travers CP et al. *JAMA Network Open*.
